# Book Notices

**Published:** 1856-07

**Authors:** 


### BOOK NOTICES.

*A Manual of the Practice of Medicine.* By GEORGE HILARO BARLOW, M.A., M.D., Cantab. Fellow of the Royal College of Physicians; Physician to Guy's Hospital, &c., &c: with additions, by D. FRANCIS CONDIE, M.D., &c., &c. Philadelphia, Blanchard & Lea, 1856.

We have received from the publishers, through Keen & Lee, of this city, the above work. From a hasty examination, we judge it to be an excellent manual, based upon the etiology and natural history of disease. It is designed for students and young practitioners, but will be found useful by those of more extensive experience and observation.

Its merit does not consist in any novelty of the principles or practice which it teaches, but in the accuracy with which disease is delineated, and the clearness and distinctness with which the principles of treatment are indicated. On the one hand, it cannot supply the place of the more elaborate works of Wood and Watson; nor, on the other, can it be ranked among the pocket editions of compends and abridgements that have undeservedly won so much popularity. The American editor has added a chapter on Cholera Infantum, one on Cerebro Spinal Meningitis, and a third on Yellow Fever. J.

---

*The Principles of Surgery.* By JAMES MILLER, F.R.S.E. & R.C.S.E., &c., &c. Fourth American, from the third and revised English edition, illustrated by two hundred and forty engravings on wood. Philadelphia, Blanchard & Lea, 1856.

It is unnecessary, we presume, to do more than to announce a new edition of "Miller's Principles." It appears without the name of an American editor, being, as the publishers tell us, an exact reprint of the author's last and revised edition.

It is, in our opinion, among the best works of the class, although by no means a full and complete treatise on the principles of surgery—indeed, we do not know of such an English or American work. J.

*The Principles and Practice of Ophthalmic Medicine and Surgery.* By T. WHARTON JONES, F.R.S., Prof. of Ophthalmic Medicine and Surgery in the University College, London, &c. With one hundred and ten illustrations. Second American edition, with additions from the second and revised London edition. Philadelphia: Blanchard & Lea, 1856.

Dr. Edward Hartshorne, of Philadelphia, is the American editor of this volume, of which it is unnecessary that we should say any thing more than to announce its appearance from the press of Blanchard & Lea. Although we have Lawrence, and Makenzie, and Wallon's works recently brought out, there was yet a demand for a work like the present, and no one perhaps could better have met the wants of the profession than T. Wharton Jones. The work is embellished with plates and illustrations, and, altogether, is an excellent guide to the student or practitioner. J.

---

*An Analytical Compendium of the various branches of Medical Science, for the Use and Examination of Students.* By JOHN NEIL, M.D., Surgeon to the Pennsylvania Hospital, &c., and FRANCIS GURNEY SMITH, M.D., Physician to St. Joseph's Hospital. A new edition, revised and improved, with three hundred and seventy-four illustrations. Philadelphia: Blanchard & Lea, 1856.

The fact that a new edition of the above work has been called for, is, we presume, evidence that it is popular with the profession. We have already had occasion to express our opinion of works of this class. They constitute at most but a bare skeleton of the subjects of which they treat, a catalogue of facts which must be already familiar to the practitioner, but for the comprehension of which the student will require the aid of more full and complete text-books. J.

---

*On Some Diseases of Women, admitting of Surgical Treatment.* By ISAAC BAKER BROWN, F.R.C.S., (by Exam.,) Surgeon Accoucheur to St. Mary's Hospital, &c., &c., illustrated by twenty-four wood engravings. Philadelphia, Blanchard & Lea, 1856.

The work is divided into two sections, the first of which treats of diseases or accidents resulting directly or indirectly from parturition; the second, of diseases or accidents of the female organs, occurring independently of pregnancy.

The first seventy-four pages are devoted to laceration and rupture of the perineum—the various methods of treatment are carefully analyzed. In the management after operation, for the purpose of constipating the bowels, allaying irritation, and controlling the inflammation, the author recommends opium, and adduces in support of his views the following judicious remarks of Dr. Handfield Jones:—

"Dr. Pereria notices the efficacy of small doses of opium (ten drops of laudanum three times a day) in such instances as the chronic or callous ulcer, the so-called varicose ulcer, in recent ulcers from wounds, in which granulation proceeds slowly, and especially in elderly persons, and in those whose constitutions have been debilitated by disease, labor, spirituous liquors, &c. 'It appears,' he says, 'to promote the most genial warmth, to give energy to the extreme arteries, and thereby maintain an equal balance of the circulation through every part of the body, and to animate the dormant energies of healthy action.'

"In the cases recorded in this paper, opium was given, not chiefly for the purpose of directly promoting the healing process, but of preventing its disturbance by mechanical and forcible disruption of the coalescing parts. For this it was freely given, and this most important end it well accomplished. But, had not this end been all-important, I own I should have feared, before trial, that the quantity of opium administered (three or four grains sometimes in a day) would have had the effect of disturbing, by its influence on the organic functions, that reparative healing process which issued in so beneficial and happy a result. For in these cases there does not appear to have been any marked asthenia, or undue irritability of the system. The terrors of surgical operations of earlier days, when the anæsthetic spell was unrevealed, may well have inflicted on the system a disturbing shock that opium alone could calm; but now there cannot be the same need for this potent agent.

"It is, however, clear, that if in these cases opium did not promote the vital healing process, at least it did not retard it: or such obstacles, as the first case presented, would not have been overcome; and the second would not have progressed so steadily and favorably. This circumstance in itself is, I think, novel and instructive.

"Perhaps, however, if we consider the matter more closely, it may appear not difficult to understand why no unfavorable, but, on the contrary, a beneficial result was produced by the opium. The condition of an ulcer, healing by granulations, may first be referred to as an extreme instance, illustrating the great waste of plasmatic material which occurs in such cases, and more or less in all that approach to it. Much of the effused plasma (effused too rapidly to be organized) is cast off as effete matter, having taken the form of pus; much is organized into the low type of the granulation structure, destined to future re-absorption. This waste is needless, nay injurious, as a drain to the system: and if it can be prevented, as sometimes it may, by applications that exclude the air; or restrained and limited, as is done by the common water-dressing, the reparative process goes on much better, and with less constitutional disturbance.

"Again, if, as in the case before us, two fresh incised surfaces are brought together, and the aim is to induce them to unite by the first intention, what can be more prejudicial to this than the effusion of much plasma, or any, the least, approach to the above-mentioned condition? To form a connecting medium across which capillaries may anastomose and fibres unite, the thinnest film of exudation is sufficient, and the thinner the better; for the organizing process is of necessity slow, far slower than the exudative; the capillary loops must take many hours to unite—the opposed fibres some days to blend, by means of the connecting material, and the further the old surfaces are separated the longer this must be delayed, and the more of the exuded matter, which itself has produced the separation, will pass into the form of effete and purulent fluid. Now, this tendency to the excessive effusion of plasma, opium very probably restrains, somewhat it may be as it restrains a flux from a mucous surface: the hurried action is stilled, the vascular excitement tending to inflammation allayed, the sedative influence of the drug assisting Nature in her work, by preventing that which would mar or delay it. The imparting of energy to the extreme arteries, which Dr. Pereira speaks of, we know from observation to be the restoration of their tonicity, enabling distended, relaxed, and conjested vessels to resume their natural calibre, and thus to transmit a due and not excessive quantity of blood in a current of proper velocity to the parts they supply. The restoration of the proper function of the arteries, 'the conductors and disposers of the blood,' as John Hunter accurately defined them, will manifestly tend greatly to prevent the excessive effusion of plasma, and thus remove at least one obstacle to the progress of reparation.

"It seems, therefore, reasonable to expect that opium, so long as it does not manifestly disorder the nervous system or the organic functions, would tend powerfully to promote the healing process, and this expectation is amply borne out by the results of the cases recorded."

The author concludes:—"1st. That the worst forms of lacerated perineum, of however long standing, may be cured by the operation. 2d. That immediately on the occurrence of the accident, it should be resorted to. 3d. That subsequent parturition is possible without injury to the restored perineum."

For vissico-vaginal fistula, the author recommends the clamp suture, used by Dr. Marion Sims, now of New York. In reference to the mode of operating, &c., there is nothing which is not already familiar to our readers.

The chapter on ovarian dropsy, or escysted dropsy of the ovary, occupies 105 pages. The questions considered, are:—The origin and structure of ovarian cysts; the formation of multilocular or proliferous cysts; the growth of cysts; the direction of growth; the intercommunication of cysts; the communication of cysts with the fallopian tubes; the contents of ovarian cysts; dropsy of fallopian tubes; the causes of ovarian dropsy; the symptoms and course of the disease; the diagnosis and signs of the disease. In reference to this last question, the following is a resume of the special signs of the disease:—

"When, with a slowly-increasing abdominal tumor, there are such general signs as emaciation, sunken or contracted features; the absence of marked œdema of the legs; of the special symptoms of ascites, or of those organic lesions productive of it; of any notable impairment of the patient's activity; of any great deterioration of the functions of life, and of the characteristic signs of pregnancy, we may suspect ovarian dropsy to exist. When percussion reveals fluctuation, and in every change of posture the fluid is detected at the most prominent part of the tumor, whilst the intestinal sound is present only on one side, and the dull sound extends into the pelvis, ovarian dropsy may be more than suspected, it may be presumed to exist.

"When, in an earlier stage, an examination *per vaginam et rectum* discovers an elastic tumor in the recto-vaginal pouch, loose in position, and probably distinctly fluctuating, without the presence of the symptoms of hernia, or of the pain of a prolapsed ovary, then we may be almost certain that it is a dropsical ovarian cyst, and, by watching, the progressive increase of the tumor strengthens the conviction. Likewise, it should be remembered, that, when there is only one cyst, the tumor is generally more perceptible on one side of the body, and its surface feels more equal; but that when there is a plurality of cysts, the unilateral character of the tumor is liable to be lost, the symptoms to be more or less obscured, and the fluctuation less distinct.

"Lastly, we must ever bear in mind the many deranged conditions of organs and functions which may be and have been confounded with encysted dropsy, and which I shall presently describe *seriatim.*"

Under this head, the author also considers the value of the microscope, the exploring needle, and the uterine sound of Dr. Simpson.

The following are enumerated as diseases likely to be mistaken for ovarian dropsy:—

"1. Retroversion and retroflexion of the uterus.
2. Tumors of the uterus: *a*, a solid; *b*, fibro-cystic.
3. Ascites.
4. Pregnancy.
5. Pregnancy, complicated with ovarian dropsy.
6. Cystic tumours of the abdomen.
7. Distended bladder.
8. Accumulation of gas in the intestines.
9. Accumulation of feces in the intestines.
10. Enlargement of the liver, spleen, or kidney, or tumors connected with these viscera.
11. Recto-vaginal hernia, and displacement of the ovary.
12. Pelvic abscess.
13. Retention of the menstrual fluid from imperforate hymen.
14. Hydrometra."

These are considered separately, and the special points of difference pointed out. The last sixty-three pages are devoted to the treatment and illustration of cases. On this subject the author seems to have no peculiar views of his own, but describes briefly the different surgical operations for the cure of the disease.

The book is well worthy of a place in the library of every physician, as well as surgeon, and we commend it to our readers.

